# Diversity of Prophage DNA Regions of *Streptococcus agalactiae* Clonal Lineages from Adults and Neonates with Invasive Infectious Disease

**DOI:** 10.1371/journal.pone.0020256

**Published:** 2011-05-25

**Authors:** Mazen Salloum, Nathalie van der Mee-Marquet, Anne-Sophie Valentin-Domelier, Roland Quentin

**Affiliations:** 1 EA 3854 Bactéries et Risque Materno-foetal, Institut Fédératif de Recherche 136 Agents Transmissibles et Infectiologie, Université François-Rabelais de Tours, UFR de Médecine, Tours, France; 2 Service de Bactériologie et Hygiène Hospitalière, Hôpital Trousseau, CHRU de Tours, Tours, France; East Carolina University, United States of America

## Abstract

The phylogenetic position and prophage DNA content of the genomes of 142 *S. agalactiae* (group-B *streptococcus*, GBS) isolates responsible for bacteremia and meningitis in adults and neonates were studied and compared. The distribution of the invasive isolates between the various serotypes, sequence types (STs) and clonal complexes (CCs) differed significantly between adult and neonatal isolates. Use of the neighbor-net algorithm with the PHI test revealed evidence for recombination in the population studied (PHI, *P* = 2.01×10^−6^), and the recombination-mutation ratio (R/M) was 6∶7. Nevertheless, the estimated R/M ratio differed between CCs. Analysis of the prophage DNA regions of the genomes of the isolates assigned 90% of the isolates to five major prophage DNA groups: A to E. The mean number of prophage DNA fragments amplified per isolate varied from 2.6 for the isolates of prophage DNA group E to 4.0 for the isolates of prophage DNA group C. The isolates from adults and neonates with invasive diseases were distributed differently between the various prophage DNA groups (*P*<0.00001). Group C prophage DNA fragments were found in 52% of adult invasive isolates, whereas 74% of neonatal invasive isolates had prophage DNA fragments of groups A and B. Differences in prophage DNA content were also found between serotypes, STs and CCs (P<0.00001). All the ST-1 and CC1 isolates, mostly of serotype V, belonged to the prophage DNA group C, whereas 84% of the ST-17 and CC17 isolates, all of serotype III, belonged to prophage DNA groups A and B. These data indicate that the transduction mechanisms, i.e., gene transfer from one bacterium to another by a bacteriophage, underlying genetic recombination in *S. agalactiae* species, are specific to each intraspecies lineage and population of strains responsible for invasive diseases in adults and neonates.

## Introduction

Group-B *streptococcus* emerged in the 1960s as a major cause of neonatal morbidity and mortality in the United States and Europe [1], [2]. Sepsis and meningitis were the most severe diseases caused by this bacterium in neonates. Three decades later, *S. agalactiae* emerged as a pathogen responsible for various infections in non pregnant adults, particularly in elderly subjects with underlying conditions [3]–[5]. Primary bacteremia is the most serious clinical syndrome reported in adults, accounting for almost 24% of all *S. agalactiae* infections in adults [6]. Meningitis is less frequent, accounting for about 4% of *S. agalactiae* infections in adults, but is associated with very high mortality rates (27% to 34%) [5], [6].

Previous studies, based on multilocus enzyme electrophoresis (MLEE), pulsed-field gel electrophoresis (PFGE), restriction digestion pattern (RDP) analysis, and multilocus sequence typing (MLST), have shown that *S. agalactiae* strains from certain phylogenetic lineages are more frequently implicated in neonatal invasive infections, such as neonatal meningitis in particular, than in adult infections [7]–[10]. Strains of other lineages have recently been found to be more specifically implicated in adult infections, particularly skin and osteoarticular infections [11].

There is growing evidence to suggest that lysogeny plays an important role in the virulence and evolution of several bacteria. Prophage-encoded virulence factors have been identified in many bacterial species, including *Vibrio cholera*, *Salmonella enterica*, *Escherchia coli*, *Clostridium botulinum*, *Corynebacterium diphtheria*, *Staphylococcus aureus* and *Streptococcus pyogenes* [12]. These virulence factors include extracellular toxins, proteins altering antigenicity or involved in invasion, enzymes and other factors. Temperate phages may also mediate the adaptation of lysogens to new hosts, thereby increasing their fitness [12]–[14]. Lysogeny also contributes to intraspecies genomic diversity in bacteria. For example, several phage-encoded proven or putative virulence factors in *S. pyogenes* species account for differences in gene content between strains [15].

In *S. agalactiae*, lysogeny was first described in 1969, when temperate phages with double-stranded DNA were isolated from strains of bovine origin [16]. Ten years later, a phage-typing system was developed and used for epidemiological investigation [17]–[21]. An analysis of sequenced *S. agalactiae* strains showed that phage-associated genes accounted for 10% of all strain-specific genes [22], [23]. *S. agalactiae* strains from different lineages, isolated from neonatal and adult patients with particular diseases have since been shown to have a greater exposure to lysogeny than colonizing strains [11], [24]. The relationships between temperate phages and *S. agalactiae* strains depend on the phage type and/or the bacterial lineage [25]. Lysogeny may therefore have affected the evolution of *S. agalactiae* species, modifying fitness and affecting adaptation to new hosts or virulence.

In this study, we used a PCR-based method recognizing *S. agalactiae* prophages to determine the diversity of prophage DNA fragments in the genome of invasive isolates from blood cultures and cerebrospinal fluid (CSF), comparing the findings for adult and neonatal patients. The genetic relationships between the prophage DNA fragments present in invasive isolate genomes were determined by hierarchical analysis. We investigated the correlations between prophage DNA content, the clinical circumstances of isolation (from neonates or adults) and the phylogenetic position of *S. agalactiae* isolates characterized by serotyping and multilocus sequence typing (MLST).

## Materials and Methods

### Bacterial isolates

We studied 142 *S. agalactiae* invasive isolates collected in various regions of France during previous epidemiological studies [26], [27]. There were 75 isolates from adults (aged 28 to 98 years; mean age, 72 years) presenting bacteremia (67 isolates isolated from blood cultures) or meningitis (8 isolates from CSF). The other 67 isolates were obtained from neonates (aged from one day to three months) presenting bacteremia (n = 20) or meningitis (n = 47).

### Serotyping

Isolates were serotyped by PCR, as previously described [28], using primers based on the sequences of the capsular polysaccharide gene clusters, and allowing to define the major GBS serotypes.

### MLST analyses

The genetic diversity and phylogenetic distribution of invasive isolates were determined by MLST, carried out as described by Jones *et al.* [10]. Comparison of the allelic sequences for seven allelic housekeeping genes allowed to define sequence types (STs). An unweighted group pair method of averages tree was drawn from allelic profile data using eBURST software (http://eburst.mlst.net/) and the entire group B streptococcus (GBS) MLST database (http://pubmlst.org/sagalactiae/), and defined CC in the species. A phylogenetic network was applied to 43 parsimonious-informative (PI) sites in SplitsTree4 (http://splitstree.org/), with the neighbor-net algorithm [29]. Recombination between isolates and STs was evaluated by calculating the pairwise homoplasy index (PHI) [30]. Recombination and mutation rates, calculated from MLST data, were evaluated with the method of Feil *et al.* [31].

### PCR for the detection of prophage DNA fragments in the genomes of *S. agalactiae* isolates

We previously identified and characterized bacteriophages and prophage remnants from *S. agalactiae* genomes and designed primer pairs recognizing prophage sequences for PCR [24], [25]. Ten of these primer pairs were used here for evaluation of the prophage DNA content of the 142 *S. agalactiae* isolates studied (Table 1). PCR was carried out with a Chromo 4 system instrument (Bio-Rad, Hercules, CA, USA). The reaction mixture had a final volume of 25 µl and contained 5 µl of extracted DNA, 0.5 µM of each primer, and IX iQ SYBR green Supermix (Qiagen SA, Courtaboeuf, France) including 3 mM MgCl_2_. The amplification program comprised 40 cycles of 10 s at 94°C, 10 s at the annealing temperature (45°C for F5, 48°C for F7, 49°C for F10, and SAK_2094, 50.5°C for SAJ_2395, and SAK_1326, 52°C for SAK_0748, or 54°C for SAG0566, SAK_2090, and SAK_0738) and 30 s at 72°C. The reaction products were then cooled to 35°C and subjected to a post-PCR melting cycle by increasing the temperature by 0.2°C for each 10-s cycle, up to 95°C.

**Table 1:** PCR primers and amplicon sizes for prophage screening.

Prophage DNA fragment	Target gene description	Reference strain(s)	PCR primer		Amplicon size (bp)
			Orientation	sequence (5′→3′)	
F5	A terminase large subunit	*S.pyogenes* 10394	ForwardReverse	ATC TTA GCA AGC TCC CAC GA TCA ACG GCT GGT ATG GAT TT	341
F7	A phage-associated cell wall hydrolase and a phage-associated lysin	*S. pyogenes* 10394SpyM6	ForwardReverse	AGG CCG CAA CCT TAA ATC T CGA GTG AAA ACG TGT CTG G	497
F10	A phage-encoded transcriptional regulator, ArpU family	*S. pyogenes* 5005	ForwardReverse	TCA GCA GAG GAA GGA AAG GA CAA TCA AAG AGC CCT CCC TA	510
SAG0566	Single-strand binding protein prophage lambda Sa1	*S. agalactiae* 2603 V/R18RS21	ForwardReverse	GTG CTT TGG TTG GAA TTA C TCT GTT GTT GGC TAT TGC	132
SAK_0738	DNA methylaseprophage lambda W4	CJB111A909	ForwardReverse	GGG ATA AGA AAG CCA ATC ACA TAG ATA GAC GCA TCG	172
SAK_0748	Phage major capsid protein HK97 family	CJB111A909	ForwardReverse	TGA TTT CTC TTA CTA CTG GAT TG CGC TTC TGG TAG AAC GAG	136
SAK_2090	BRO domain protein, prophage antirepressorprophage Sa05	A909H36BCJB111	ForwardReverse	TAG AGC ACC AAG GCG AAT G AAA CGA CCT CAT CAA CTA AAC G	102
SAK_2094	Prophage Sa05site-specific recombinasephage integrase family	A909H36BCJB11118RS21COH1	ForwardReverse	AAA GAG TAA AGC ATT TCG CCT AAT CTA TAT TGG AGT TC	526
SAJ_2395	Phage terminase-like protein, large subunit (remnant)	18RS21515	ForwardReverse	TGA TAG ATA AGT ATG TGA GAT TC TTG TCT TTC CGA GTT AGC	251
SAK_1326	Site-specific recombinase, phage integrase family (remnant)	A909H36BCJB111	ForwardReverse	TTT GAC CTA CGG GAT TAT G TGA ACG CCA TCT TAG AAG	261

The genetic relationships between the prophage DNA regions of the genomes of the isolates studied were investigated by a hierarchical analysis based on the Jaccard dichotomy coefficient method, as implemented in SYSTAT 12 software.

### Statistical analysis

Data were analyzed by chi-squared tests and Fisher's exact tests, to evaluate associations, with *P* values≤0.05 considered significant.

## Results

### Serotyping

One isolate from the blood culture of an adult patient could not be typed. The serotype distribution of isolates from adults did not differ as a function of the disease: bacteremia or meningitis (Table 2; *P* = 0.97). Similarly, no difference in serotype distribution between bacteremia and meningitis was observed for isolates from neonates (table 2; *P* = 0.41).

**Table 2:** Serotypes of *S. agalactiae* isolates from blood cultures and CSF from adults and neonates.

Serotype	No. of *S. agalactiae* strains (% prevalence) from					
	Adult			Neonate		
	Blood culture	CSF	Total	Blood culture	CSF	Total
Ia	15 (22)	2 (25)	17 (23)	4 (20)	4 (9)	8 (12)
Ib	12 (18)	2 (25)	14 (19)	-	3 (6)	3 (4)
II	4 (6)	-	4 (5)	-	-	-
III	12 (18)	2 (25)	14 (19)	15 (75)	38 (81)	53 (79)
IV	2 (3)	-	2 (3)	-	-	-
V	21 (31)	2 (25)	23 (31)	1 (5)	2 (4)	3 (4)
NT^a^	1 (1)	-	1 (1)	-	-	-
Total	67	8	75	20	47	67

a NT, Nontypable.

By contrast, the serotype distributions of isolates from adults and neonates suffering from invasive disease differed significantly (Table 2; *P*<0.00001). Indeed, 79% of the invasive *S. agalactiae* isolates from neonates belonged to serotype III, whereas invasive isolates from adults belonged to four major serotypes: serotypes V (31%), Ia (23%), Ib (19%), and III (19%) (Table 2).

### Genetic diversity, recombination and mutation of housekeeping genes

**Figure 1:**
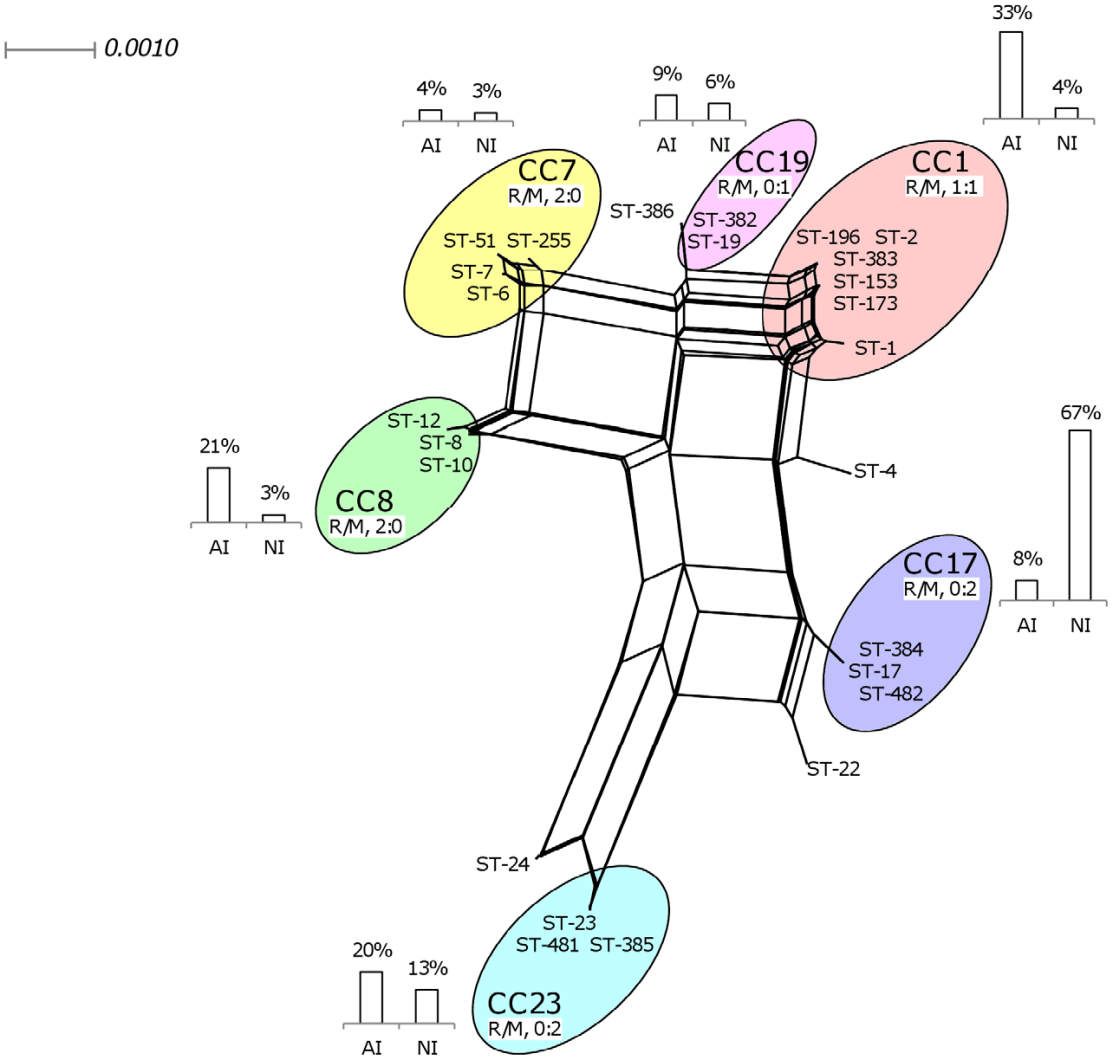
Genetic diversity and sequence type (ST) distribution, determined by MLST [**10**], of 142 *S. agalactiae* isolates from cases of adult (AI) and neonatal (NI) invasive disease. We show the phylogenetic network applied to 43 parsimonious-informative sites from a total of 3,456 nucleotides generated with the neighbour-net algorithm for the 142 strains studied (http://splitstree.org/) [29]. Strains were grouped into clonal complexes (CCs) with eBURST software (http://eburst.mlst.net/). Columns indicate the percentages of AI and NI strains in each CC. Recombination (R) and mutation (M) rates, based on MLST data, were evaluated as described by Feil *et al.* [31]. The estimated recombination-mutation ratio (R/M) varied as a function of the CC to which the strain belonged.

MLST identified 25 different sequence types (STs) among the 142 invasive isolates tested (Fig. 1). eBURST software assigned 137 isolates from 21 STs to six major clonal complexes (CCs): CC1 (28 isolates), CC7 (5 isolates), CC8 (18 isolates), CC17 (51 isolates), CC19 (11 isolates), and CC23 (24 isolates). The isolates of each serotype were distributed between several STs, but this distribution was not random (Table 3; *P*<0.00001). The isolates of serotypes Ia, III, IV, and V were mostly associated with ST-23 (80%), ST-17 (73%), ST-196 (100%) and ST-1 (81%), respectively. Similarly, the isolates of serotypes Ia, Ib, III, and IV+V mostly belonged to CC23 (84%), CC8 (88%), CC17 (76%), and CC1 (96%), respectively.

**Table 3:** Serotype of *S. agalactiae* isolates from the various STs and CCs implicated in adult and neonatal invasive infections.

CC	ST	No. (%) of isolates according to serotype						
(No. of isolates)	(No. of isolates)	Ia	Ib	II	III	IV	V	NT^a^
1 (28)				1 (25)		2 (100)	25 (96)	
	1 (22)			1 (25)			21 (81)	
	2 (1)						1 (4)	
	153 (1)						1 (4)	
	173 (1)						1 (4)	
	196 (2)					2 (100)		
	383 (1)						1 (4)	
7 (5)		2 (8)	2 (12)		1 (1)			
	6 (1)		1 (6)					
	7 (2)	1 (4)			1 (1)			
	51 (1)	1 (4)						
	255 (1)		1 (6)					
8 (18)			15 (88)	2 (50)			1 (4)	
	8 (8)		8 (47)					
	10 (5)		3 (18)	1 (25)			1 (4)	
	12 (5)		4 (24)	1 (25)				
17 (51)					51 (76)			
	17 (49)				49 (73)			
	384 (1)				1 (1)			
	482 (1)				1 (1)			
19 (11)					10 (15)			1 (100)
	19 (10)				9 (13)			1 (100)
	382 (1)				1 (1)			
23 (24)		21 (84)			3 (4)			
	23 (22)	20 (80)			2 (3)			
	385 (1)	1 (4)						
	481 (1)				1 (1)			
Singletons (5)		2 (8)		1 (25)	2 (3)			
	4 (1)	1 (4)						
	22 (2)				2 (3)			
	24 (1)	1 (4)						
	386 (1)			1 (25)				
Total		25	17	4	67	2	26	1

a NT, nontypable.

The distribution of isolates from adults between STs did not differ according to the disease: bacteremia or meningitis (Table 4; *P* = 0.87). Similarly, the distribution of isolates from neonates between STs did not depend on the whether the child had bacteremia or meningitis (table 4; *P* = 0.25). By contrast, the distribution of invasive isolates among STs differed significantly between isolates from neonates and those from adults (Table 4; *P*<0.00001). Indeed, the 67 invasive isolates from neonates belonged to only 11 STs, whereas the 75 isolates from adults were more diverse, belonging to 21 STs (Table 4). In addition, 44 of the 67 invasive isolates from neonates (66%) belonged to ST-17, whereas isolates from adults were significantly more frequently associated with ST-1 (19/75; 25%) and ST-23 (14/75; 19%) (Table 4; *P*<0.00001).

**Table 4:** CC, ST of *S. agalactiae* isolates from adult and neonatal invasive infections.

CC	ST	No. (%) of isolates from					
(No. of isolates)	(No. of isolates)	Adult			Neonate		
		Blood culture	CSF	Total	Blood culture	CSF	Total
1 (28)		23 (34)	2 (25)	25 (33)	1 (5)	2 (4)	3 (4)
	1 (22)	18 (27)	1 (13)	19 (25)	1 (5)	2 (4)	3 (4)
	2 (1)		1 (13)	1 (1)			
	153 (1)	1 (1)		1 (1)			
	173 (1)	1 (1)		1 (1)			
	196 (2)	2 (3)		2 (3)			
	383 (1)	1 (1)		1 (1)			
7 (5)		3 (4)		3 (4)		2 (4)	2 (3)
	6 (1)					1 (2)	1 (1)
	7 (2)	1 (1)		1 (1)		1 (2)	1 (1)
	51 (1)	1 (1)		1 (1)			
	255 (1)	1 (1)		1 (1)			
8 (18)		14 (21)	2 (25)	16 (21)		2 (4)	2 (3)
	8 (8)	6 (9)		6 (8)		2 (4)	2 (3)
	10 (5)	4 (6)	1 (13)	5 (7)			
	12 (5)	4 (6)	1 (13)	5 (7)			
17 (51)		5 (7)	1 (13)	6 (8)	13 (65)	32 (68)	45 (67)
	17 (49)	4 (6)	1 (13)	5 (7)	12 (60)	32 (68)	44 (66)
	384 (1)	1 (1)		1 (1)			
	482 (1)				1 (5)		1 (1)
19 (11)		6 (9)	1 (13)	7 (9)		4 (9)	4 (6)
	19 (10)	5 (7)	1 (13)	6 (8)		4 (9)	4 (6)
	382 (1)	1 (1)		1 (1)			
23 (24)		13 (19)	2 (25)	15 (20)	5 (25)	4 (9)	9 (13)
	23 (22)	12 (18)	2 (25)	14 (19)	4 (20)	4 (9)	8 (12)
	385 (1)	1 (1)		1 (1)			
	481 (1)				1 (5)		1 (1)
Singletons (5)		3 (4)		3 (4)	1 (5)	1 (2)	2 (3)
	4 (1)	1 (1)		1 (1)			
	22 (2)	1 (1)		1 (1)	1 (5)		1 (1)
	24 (1)					1 (2)	1 (1)
	386 (1)	1 (1)		1 (1)			
Total		67	8	75	20	47	67

As for STs, the distribution of isolates from adults between CCs did not depend on the nature of the disease (table 4; *P* = 0.99), and this was also the case for isolates from neonates (Table 4; *P* = 0.25). The distribution of invasive isolates among the various CCs differed significantly between isolates of neonatal and adult origin (Table 4; *P*<0.00001). Indeed, neonatal invasive isolates most frequently belonged to CC17 (45/67; 67%), whereas isolates from adults with invasive disease mostly belonged to CC1 (25/75; 33%), CC8 (16/75; 21%), and CC23 (15/75; 20%).

We identified a total of 55 variable nucleotide sites in the 3,456 bp of concatenated gene sequences, and 43 sites were parsimonious informative (PI), e.g. informative positions. There were 41 PI sites in neonatal isolates and 43 PI sites in adult invasive isolates. The phylogenetic network applied to the PI sites from the 3,456 total nucleotides with the neighbour-net algorithm for all isolates studied is shown in Figure 1. The overall level of genetic diversity among the invasive isolates was estimated by SplitsTree4 software at 0.015 for adult invasive isolates and 0.013 for neonatal invasive isolates.

Use of the neighbour-net algorithm with the PHI test revealed evidence for recombination in the population studied (PHI, *P* = 2.01×10^−6^; Fig. 1). The method of Feil *et al.* [31] was applied to the six major CCs defined by eBURST (Table 5), resulting in the identification of 13 single locus variants (SLV) of the six major founder STs. Five of these SLVs displayed allelic polymorphism common to the STs of various CCs. Based on the data shown in table 5 and applying the theory of Feil *et al.* to our population of 142 isolates, the rate of homologous recombination events was evaluated at six for every seven point mutations (recombination-mutation ratio of 6∶7), thus confirming the results of the PHI test suggesting a high level of homologous recombination within the *S. agalactiae* population studied. The estimated recombination-mutation ratio (R/M) differed between CCs: the isolates of CCs 7 and 8 presented no evidence of mutation (R/M, 2∶0), whereas those of CCs 17, 19, and 23 displayed no evidence of recombination (R/M, 0∶2 or 0∶1), and CC1 isolates displayed equal frequencies of recombination and mutation (1∶1) (Table 5; Fig. 1).

**Table 5:** Distribution of the SLVs of the major clonal complexes as a function of difference in the number of nucleotides (one or more) with respect to the sequence of the founder sequence type.

Clonal complex	Allelic profile MLST								No. of SLVs differing at a single-nucleotide site		No. of SLVs differing at multiple-nucleotide sites	
	ST	adhP	pheS	atr	glnA	sdhA	glck	tkt	Different	Shared	Different	Shared
CC1												
Founder ST	1	1	1	2	1	1	2	2				
SLV	2	1	1	3	1	1	2	2	0	1	0	0
	153	36	1	2	1	1	2	2	1	0	0	0
	173	38	1	2	1	1	2	2	1	0	0	0
	383	1	1	2	42	1	2	2	0	1	0	0
Sub-totals									2	2	0	0
CC7												
Founder ST	7	10	1	2	1	3	2	2				
SLV	6	9	1	2	1	3	2	2	0	1	0	0
	51	10	1	3	1	3	2	2	0	1	0	0
Sub-totals									0	2	0	0
CC8												
Founder ST	8	4	1	4	1	3	3	2				
SLV	10	9	1	4	1	3	3	2	0	1	0	0
	12	10	1	4	1	3	3	2	0	0	0	1
Sub-totals									0	1	0	1
CC17												
Founder ST	17	2	1	1	2	1	1	1				
SLV	384	2	1	1	2	1	37	1	1	0	0	0
	482	2	1	1	2	47	1	1	1	0	0	0
Sub-totals									2	0	0	0
CC19												
Founder ST	19	1	1	3	2	2	2	2				
SLV	382	72	1	3	2	2	2	2	1	0	0	0
Sub-totals									1	0	0	0
CC23												
Founder ST	23	5	4	6	3	2	1	3				
SLV	385	5	4	6	3	2	1	30	1	0	0	0
	481	5	4	6	51	2	1	3	1	0	0	0
Sub-totals									2	0	0	0
totals									7	5	0	1

### Prophage DNA content in invasive *S. agalactiae* isolate genomes

**Figure 2:**
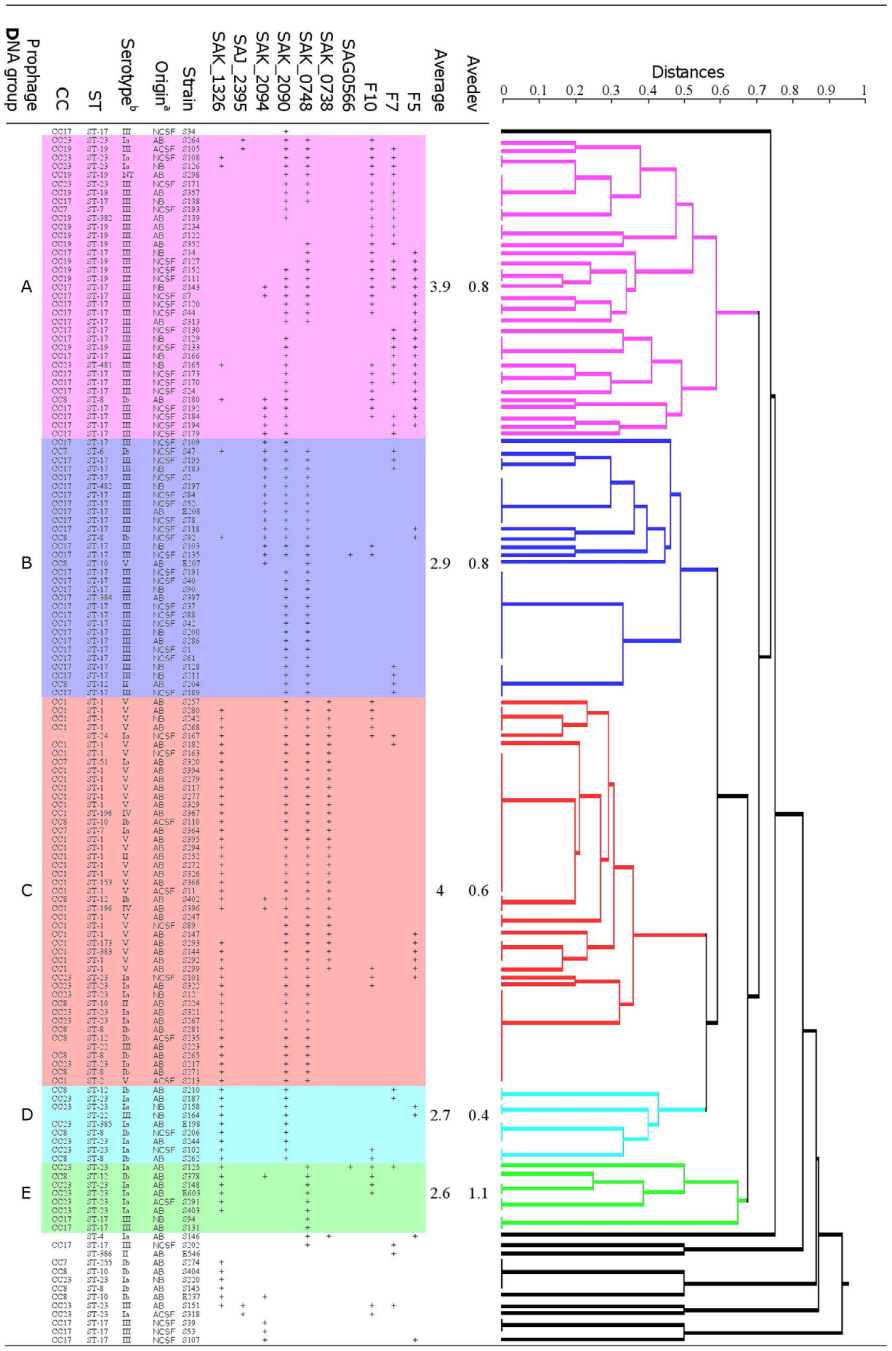
Distribution of 141 *S. agalactiae* isolates from adult (ACSF and AB) and neonatal (NCFS and NB) patients with invasive disease between prophage DNA groups, on the basis of PCR evaluations of the prophage content of isolates. Jaccard analysis generated a dendrogram of similarity values for the 10 prophage sequences described in table 1 (SYSTAT 12 software). Five major prophage DNA groups were defined (groups A to E). The mean number of prophage DNA fragments amplified from strains by PCR and the mean number of absolute deviations (Avedev) were calculated for each prophage DNA group. ^a^ anatomic origin of isolates; ^b^ serotype of isolates; ST, sequence-type; CC, clonal complex; NT, nontypeable.

None of the prophage DNA fragments studied was detected by PCR in one isolate from a CSF sample from an adult. For each of the remaining 141 isolates, PCR amplified one to six of the 10 prophage DNA fragments studied. The genetic relationships between the prophage DNA regions of isolate genomes were plotted as a dendrogram (Fig. 2). This analysis assigned 90% of the isolates (127/141) to five major prophage DNA groups: A to E. The remaining 14 isolates had distantly related prophage DNA regions.

The natures and frequencies of the prophage DNA fragments amplified from the isolates differed significantly between prophage DNA groups (Table 6; *P*<0.00001). The mean number of prophage DNA fragments amplified per isolate varied from 2.6 in prophage DNA group E to 4.0 in prophage DNA group C. The amplification patterns and diversity of prophage DNA fragments observed differed considerably within prophage DNA groups (Table 6). For the isolates of prophage DNA groups A and C, eight of the 10 prophage targets studied were found in at least one isolate and PCR amplified a large number of prophage DNA fragments from each isolate (means of 3.9 and 4, respectively; Fig. 2). Nevertheless, the diversity of prophage DNA amplification patterns was greater in group A than in group C. Indeed, 24 different prophage patterns were observed for the 35 isolates in group A, whereas only 13 prophage patterns were obtained for the 45 isolates in group C. In addition, the maximal distance between prophage patterns was 0.58 in group A and 0.35 in group C (Fig. 1). The mean number of prophage DNA fragments amplified per isolate was lower (2.6 to 2.9; Fig. 2) for the three remaining prophage DNA groups, B, D and E. Pattern diversity was lowest in group B (11 prophage patterns for 30 isolates), and slightly greater in groups D (4 patterns for 9 isolates) and E (6 patterns for 8 isolates).

**Table 6:** Distribution of the *S. agalactiae* isolates of various origins, serotypes, sequence types, and clonal complexes between prophage DNA groups, as displayed by SYSTAT 12 software^a^.

Characteristic(No. of strains)		No. of strains (%) per prophage DNA group					
	NP	A (35)	B (30)	C (45)	D (9)	E (8)	minor groups (14)
Prophage DNA fragment							
F5	109 (77)	21 (15)	2 (1)	6 (4)	2 (1)		2 (1)
F7	101 (71)	26 (18)	7 (5)	2 (1)	2 (1)	1 (<1)	3 (2)
F10	96 (68)	28 (20)	2 (1)	8 (6)	2 (1)	4 (3)	2 (1)
SAG0566	140 (99)		1 (<1)			1 (<1)	
SAK_0738	109 (77)			32 (23)			1 (<1)
SAK_0748	40 (28)	18 (13)	29 (20)	45 (32)		8 (6)	2 (1)
SAK_2090	29 (20)	29 (20)	29 (20)	45 (32)	9 (6)		1 (<1)
SAK_2094	113 (80)	7 (5)	15 (11)	2 (1)		1 (<1)	4 (3)
SAJ_2395	138 (97)	2 (1)					2 (1)
SAK_1326	74 (52)	4 (3)	2 (1)	41 (29)	9 (6)	6 (4)	6 (4)
Origin							
Adult blood culture (67)		9 (13)	5 (7)	35 (52)	5 (7)	6 (9)	7 (10)
Adult CSF (8)	1 (13)	1 (13)		4 (50)		1 (13)	1 (13)
Adult (75)	1 (1)	10 (13)	5 (7)	39 (52)	5 (7)	7 (9)	8 (11)
Neonatal blood culture (20)		7 (35)	7 (35)	2 (10)	2 (10)	1 (5)	1 (5)
Neonatal CSF (47)		18 (38)	18 (38)	4 (9)	2 (4)		5 (11)
Neonate (67)		25 (37)	25 (37)	6 (9)	4 (6)	1 (1)	6 (9)
Serotype							
Ia (25)		3 (12)		9 (36)	5 (20)	5 (20)	3 (12)
Ib (17)		1 (6)	2 (12)	6 (35)	3 (18)	1 (6)	4 (24)
II (4)			1 (25)	2 (50)			1 (25)
III (67)	1 (1)	30 (45)	26 (39)	1 (1)	1 (1)	2 (3)	6 (9)
IV (2)				2 (100)			
V (26)			1 (4)	25 (96)			
NT (1)		1 (100)					
Sequence type							
1 (22)				22 (100)			
8 (8)		1 (13)	1 (13)	3 (38)	2 (25)		1 (13)
10 (5)			1 (20)	2 (40)			2 (40)
12 (5)			1 (20)	2 (40)	1 (20)	1 (20)	
17 (49)	1 (2)	17 (35)	24 (49)			2 (4)	5 (10)
19 (10)		10 (100)					
23 (22)		4 (18)		6 (27)	4 (18)	5 (23)	3 (14)
Others (21)		3 (14)	3 (14)	10 (48)	2 (10)		3 (14)
Clonal complex							
1 (28)				28 (100)			
7 (5)		1 (20)	1 (20)	2 (40)			1 (20)
8 (18)		1 (6)	3 (17)	7 (39)	3 (17)	1 (6)	3 (17)
17 (51)	1 (2)	17 (33)	26 (51)			2 (4)	5 (10)
19 (11)		11 (100)					
23 (24)		5 (21)		6 (25)	5 (21)	5 (21)	3 (13)
Others (5)				2 (40)	1 (20)		2 (40)

a NP no prophage amplification; NT, nontypable.

Each prophage DNA group was characterized by the presence of particular prophage DNA regions in the isolate genomes (Table 6). Prophage DNA group A was characterized by the frequent amplification of prophage DNA fragments F5, F7, F10, SAK_0748 and SAK_2090; prophage DNA group B was characterized by the frequent amplification of prophage DNA fragments SAK_0748, SAK_2090, and SAK_2094; prophage DNA group C was characterized by the frequent amplification of prophage DNA fragments SAK_0738, SAK_0748, SAK_2090, and SAK_1326; prophage DNA group D was characterized by the frequent amplification of prophage DNA fragments SAK_2090 and SAK_1326 and prophage DNA group E was characterized by the frequent amplification of prophage DNA fragments SAK_0748 and SAK_1326.

The distribution of isolates between prophage DNA groups did not depend on disease (bacteremia or meningitis) for either adult (Table 6; *P* = 0.86) or neonatal (Table 6; *P* = 0.54) isolates. By contrast, isolates from adults and neonates with invasive diseases displayed significantly different distributions among prophage DNA groups (Table 6; *P*<0.00001). Indeed, 39 of the 75 (52%) adult isolates belonged to group C, whereas 50 of the 67 (74%) neonatal isolates were evenly distributed between two groups, A and B.

The distribution of isolates from the various serotypes, STs and CCs between prophage DNA groups was not random (Table 6, Fig. 2) (P<0.00001). Isolates from the two major lineages, ST-1 and ST-17, and their corresponding clonal complexes, CC1 and CC17, frequently implicated in adult and neonatal invasive diseases, respectively, had particular prophage DNA contents. All 22 ST-1 and 28 CC1 isolates (100%), most of which were of serotype V, belonged to prophage DNA group C. Forty-one of the 49 ST-17 isolates (84%) and 43 of the 51 CC17 isolates (84%), all of serotype III, belonged to groups A and B. Remarkably, the 10 invasive isolates of ST-19 and 11 invasive isolates of CC19, which rarely cause invasive disease in adults and neonates, also clustered with group A (Fig. 2). Thus, invasive ST-19/CC19 isolates had a prophage DNA content similar to that of CC17 isolates capable of invading the CSF of neonates [10], [32]–[36]. The isolates of ST-8 and ST-23 and the corresponding clonal complexes, CC8 and CC23, frequently implicated in adult invasive disease, were mostly of serotype Ib and Ia, respectively, and were distributed between the five major prophage DNA groups, indicating considerable diversity in terms of prophage DNA content.

## Discussion

*Streptococcus agalactiae* is a well known cause of sepsis and meningitis in neonates and is now recognized as a non exceptional cause of bacteremia and meningitis in adults. Nevertheles, *S. agalactiae* isolates responsible for causing disease in adults have not been much studied yet. Prophage genes account for 10% of all strain-specific genes in *S. agalactiae*, but few studies have investigated the prophage DNA content of the *S. agalactiae* genome. Given that temperate phages can mediate the adaptation of bacteria to new ecological conditions and affect the evolution and pathogenic power of individuals (12), we studied the diversity of prophage DNA regions in the genomes of *S. agalactiae* isolates from various phylogenetic lineages obtained from adults and neonates with invasive infections. The *S. agalactiae* isolates implicated in bacteremia and meningitis displayed considerable genetic diversity, due to a number of different genetic events, including mutation and recombination (PHI, *P* = 2.01×10^−6^; Fig. 1 and recombination-mutation ratio of 6∶7), and were exposed to particular transduction mechanisms in gene recombination (Table 6, Fig. 2). Nevertheless, our data indicate that the prophage DNA content of isolates resulting in lysogeny were specific to each intraspecies lineage of isolates, suggesting a possible contribution to the differentiation of the species into clones, with each clone differing markedly from the others in terms of its propensity to cause invasive disease in adults or neonates.

CC17 isolates have been well characterized and are clearly associated with invasive diseases in neonates (Fig. 1, Table 3; [10], [32]–[36]). The invasive CC17 isolates studied were fairly homogeneous in terms of their recombination levels (Fig. 1), consistent with recent findings [36]. By contrast, the CC17 isolates displayed considerable diversity in terms of their prophage DNA content. Indeed, the prophage DNA fragments found in their genomes belonged to two different, distant prophage DNA groups (A and B), each characterized by a particular profile of prophage DNA fragments (Fig. 2). *S. agalactiae* may be considered a model of bacteria subjected to temporal changes in habitat, for which prophage–bacterial interaction has been an essential survival strategy during evolution, for both the prophage and the bacterium. We can hypothesize that the two clades of CC17 isolates, defined on the basis of their prophage DNA content, may result from adaptation, at different times, to two markedly different ecological systems, such as the bovine or human intestinal or genital tracts, with very different phage constituents [12], [14], [37], [38]. These two prophage DNA content-based clades in CC17 are both capable of invading the central nervous system of neonates. However, the virulence mechanisms of these two clades may differ considerably, given that the transduction mechanisms in gene recombination may affect bacterial virulence.

Since the mid-1990s, *S. agalactiae* has increasingly been considered as a pathogen responsible for various infections in adults [3], [4], [6], [39]–[41]. This change in host coincided with the emergence of a particular serotype, serotype V, which was subsequently shown to belong to a new phylogenetic lineage, CC-1 [42], [43]. In this study, we found that most of the isolates responsible for sepsis and meningitis in adults belonged to CC1 and CC8, two clones phylogenetically different from that associated with invasive infections in neonates (CC17) and with genomes containing specific prophage DNA fragments (Fig. 1; Fig. 2). This suggests that the bacteria involved in invasive infections in adults and neonates emerged and evolved in very different ways. These findings may be explained partly by the constraints linked to adaptation to different hosts. Indeed, CC17 strains have been associated with vaginal colonization, whereas the strains of STs 1 and 8 are more frequently associated with throat and anal margin carriage [44]. Bacteriophages are responsible for niche-specific horizontal gene transfer, which plays a role in structuring bacterial communities [45], [46]. The adaptation of strains to different ecological systems in humans may therefore result from different lysogeny events with different consequences. Nevertheless, the precise impact of lysogeny, which results in the adaptation of *S. agalactiae* to very different systems (the mouth, the gut, the vagina, the skin, the udder of cattle, fish or marine environments) with contrasting biological, physical and chemical properties, remains to be elucidated.

Our results showed that the invasive CC1, CC8 and CC23 isolates responsible for invasive diseases in adults were fairly homogeneous in terms of their prophage DNA content. Indeed, most of these isolates possessed the same prophage DNA fragments, those of the homogeneous prophage DNA group C, which had the highest mean number of prophage DNA fragments per isolate (mean = 4.0; Table 6, Fig. 2). The prophage DNA fragments identified in this group have previously been found to be: i) associated with CC1 and CC23 isolates responsible for adult skin and osteoarticular infections and ii) different in terms of the nature and number of fragments per isolates from those of colonizing strains [11]. These results suggest a possible link between the phenomena of lysogeny and the ability of strains to induce disease in adults, but the mechanisms involved remain unclear. As previously described in *S. aureus* and *E. coli*, lysogeny may inactivate some bacterial genes, thereby altering the levels of transcription and expression of some virulence genes [47]–[49]. Alternatively, lysogeny may facilitate the importation of new virulence genes, as described in several species, such as *S. aureus*, *S. pyogenes*, *V. cholerae* or enteric bacteria [12]. In *S. agalactiae*, the mechanism involved is likely to be more complex, as no virulence gene associated with a phage has yet been identified. It is possible that, as recently shown for the integration of other mobile genetic elements into intergenic regions [50], prophage DNA integration into the *S. agalactiae* genome modified the expression of certain factors already present in the genome. All but one of the CC8 isolates contained the SAK_1326 fragment, which was found in most CC1 isolates and all but two of the CC23 isolates (Fig. 2). This fragment is located in the genomic island harboring the *lmb* and *scpB* genes (strain NEM316; ST-23) and the fibrinogen-binding protein genes (strain H36B; ST-6, serotype Ib), which encode virulence factors known to be involved in adhesion and immune evasion. It was therefore not possible to exclude the possibility that the integration of such prophage DNA fragments into the strain genome modulates metabolism and/or the expression of virulence genes. Very little is currently known about these mechanisms. It is unclear, for example, whether they are related to the import of additional promoters, or consequences of the action of non-coding RNA or of short palindromic sequences, such as clusters of regularly interspaced short palindromic repeats, directly related to lysogeny. Future studies will focus on these phenomena.

## References

[1] Anthony BF, Okada DM (1977). The emergence of group B streptococci in infections of the newborn infant.. Annu Rev Med.

[2] Eickhoff TC, Klein JO, Daly AK, Ingall D, Finland M (1964). Neonatal sepsis and other infections due to group B beta-hemolytic streptococci.. N Engl J Med.

[3] Farley MM, Harvey RC, Stull T, Smith JD, Schuchat A (1993). A population-based assessment of invasive disease due to group B *Streptococcus* in nonpregnant adults.. N Engl J Med.

[4] Schwartz B, Schuchat A, Oxtoby MJ, Cochi SL, Hightower A (1991). Invasive group B streptococcal disease in adults. A population-based study in metropolitan Atlanta.. JAMA.

[5] Farley MM (2001). Group B streptococcal disease in nonpregnant adults.. Clin Infect Dis.

[6] Sendi P, Johansson L, Norrby-Teglund A (2008). Invasive group B Streptococcal disease in non-pregnant adults: a review with emphasis on skin and soft-tissue infections.. Infection.

[7] Takahashi S, Adderson EE, Nagano Y, Nagano N, Briesacher MR (1998). Identification of a highly encapsulated, genetically related group of invasive type III group B streptococci.. J Infect Dis.

[8] Quentin R, Huet H, Wang FS, Geslin P, Goudeau A (1995). Characterization of Streptococcus agalactiae strains by multilocus enzyme genotype and serotype: identification of multiple virulent clone families that cause invasive neonatal disease.. J Clin Microbiol.

[9] Rolland K, Marois C, Siquier V, Cattier B, Quentin R (1999). Genetic features of *Streptococcus agalactiae* strains causing severe neonatal infections, as revealed by pulsed-field gel electrophoresis and hylB gene analysis.. J Clin Microbiol.

[10] Jones N, Bohnsack JF, Takahashi S, Oliver KA, Chan MS (2003). Multilocus sequence typing system for group B streptococcus.. J Clin Microbiol.

[11] Salloum M, van der Mee-Marquet N, Domelier AS, Arnault L, Quentin R (2010). Molecular characterization and prophage DNA contents of *Streptococcus agalactiae* strains isolated from adult skin and osteoarticular infections.. J Clin Microbiol.

[12] Brussow H, Canchaya C, Hardt WD (2004). Phages and the evolution of bacterial pathogens: from genomic rearrangements to lysogenic conversion.. Microbiol Mol Biol Rev.

[13] Boyd EF, Brussow H (2002). Common themes among bacteriophage-encoded virulence factors and diversity among the bacteriophages involved.. Trends Microbiol.

[14] Chibani-Chennoufi S, Bruttin A, Dillmann ML, Brussow H (2004). Phage-host interaction: an ecological perspective.. J Bacteriol.

[15] Banks DJ, Beres SB, Musser JM (2002). The fundamental contribution of phages to GAS evolution, genome diversification and strain emergence.. Trends Microbiol.

[16] Russell H, Norcross NL, Kahn DE (1969). Isolation and characterization of *Streptococcus agalactiae* bacteriophage.. J Gen Virol.

[17] Stringer J (1980). The development of a phage-typing system for group-B streptococci.. J Med Microbiol.

[18] Anthony BF, Okada DM, Hobel CJ (1979). Epidemiology of the group B *Streptococcus*: maternal and nosocomial sources for infant acquisitions.. J Pediatr.

[19] Band JD, Clegg HW, Hayes PS, Facklam RR, Stringer J (1981). Transmission of group B streptococci traced by use of multiple epidemiologic markers.. Am J Dis Child.

[20] Boyer KM, Vogel LC, Gotoff SP, Gadzala CA, Stringer J (1980). Nosocomial transmission of bacteriophage type 7/11/12 group B streptococci in a special care nursery.. Am J Dis Child.

[21] Noya FJ, Rench MA, Metzger TG, Colman G, Naidoo J (1987). Unusual occurrence of an epidemic of type Ib/c group B streptococcal sepsis in a neonatal intensive care unit.. J Infect Dis.

[22] Tettelin H, Masignani V, Cieslewicz MJ, Eisen JA, Peterson S (2002). Complete genome sequence and comparative genomic analysis of an emerging human pathogen, serotype V *Streptococcus agalactiae*.. Proc Natl Acad Sci USA.

[23] Tettelin H, Masignani V, Cieslewicz MJ, Donati C, Medini D (2005). Genome analysis of multiple pathogenic isolates of *Streptococcus agalactiae*: implications for the microbial “pan-genome”.. Proc Natl Acad Sci USA.

[24] van der Mee-Marquet N, Domelier AS, Mereghetti L, Lanotte P, Rosenau A (2006). Prophagic DNA fragments in *Streptococcus agalactiae* strains and association with neonatal meningitis.. J Clin Microbiol.

[25] Domelier AS, van der Mee-Marquet N, Sizaret PY, Héry-Arnaud G, Lartigue MF (2009). Molecular characterization and lytic activities of *Streptococcus agalactiae* bacteriophages and determination of lysogenic strain features.. J Bacteriol.

[26] Domelier AS, van der Mee-Marquet N, Arnault L, Mereghetti L, Lanotte P (2008). Molecular characterization of erythromycin-resistant *Streptococcus agalactiae* strains.. J Antimicrob Chemother.

[27] Quentin R, Loulergue J, Mala L, Porcheron A, Grasmick C (2006). Group B streptococcus as human pathogen: origin of isolates and antibiotic susceptibility.. BEH.

[28] Kong F, Gowan S, Martin D, James G, Gilbert GL (2002). Serotype identification of group B streptococci by PCR and sequencing.. Journal of Clinical Microbiology.

[29] Huson DH, Bryant D (2006). Application of phylogenetic networks in evolutionary studies.. Mol Biol Evol.

[30] Bruen TC, Philippe H, Bryant D (2006). A simple and robust statistical test for detecting the presence of recombination.. Genetics.

[31] Feil EJ, Smith JM, Enright MC, Spratt BG (2000). Estimating recombinational parameters in *Streptococcus pneumonia* from multilocus sequence data.. Genetics.

[32] Bisharat N, Jones N, Marchaim D, Block C, Harding RM (2005). Population structure of group B *Streptococcus* from a low-incidence region for invasive neonatal disease.. Microbiology.

[33] Jones N, Oliver KA, Barry J, Harding RM, Bisharat N (2006). Enhanced invasiveness of bovine-derived neonatal sequence type 17 group B *Streptococcus* is independent of capsular serotype.. Clin Infect Dis.

[34] Luan SL, Granlund M, Sellin M, Legegard T, Spratt BG (2005). Multilocus sequence typing of Swedish invasive group B *Streptococcus* isolates indicates a neonatally associated genetic lineage and capsule switching.. J Clin Microbiol.

[35] Lin FY, Whiting A, Adderson E, Takahashi S, Dunn DM (2006). Phylogenetic lineages of invasive and colonizing strains of serotype III group B streptococci from neonates: a multicenter prospective study.. J Clin Microbiol.

[36] Manning SD, Springman AC, Lehotzky E, Lewis MA, Whittam TS (2009). Multilocus sequence types associated with neonatal group B streptococcal sepsis and meningitis in Canada.. J Clin Microbiol.

[37] Bisharat N, Crook DW, Leigh J, Harding RM, Ward PN (2004). Hyperinvasive neonatal group B *Streptococcus* has arisen from a bovine ancestor.. J Clin Microbiol.

[38] Héry-Arnaud G, Bruant G, Lanotte P, Brun S, Picard B (2007). Mobile genetic elements provide evidence for a bovine origin of clonal complex 17 of *Streptococcus agalactiae*.. Appl Environ Microbiol.

[39] Blancas D, Santin M, Olmo M, Alcaide F, Carratala J (2004). Group B streptococcal disease in nonpregnant adults: incidence, clinical characteristics, and outcome.. Eur J Clin Microbiol Infect Dis.

[40] Phares CR, Lynfield R, Farley MM, Mohle-Boetani J, Harrison LH (2008). Epidemiology of invasive group B streptococcal disease in the United States, 1999–2005.. JAMA.

[41] Skoff TH, Farley MM, Petit S, Craig AS, Schaffner W (2009). Increasing burden of invasive group B streptococcal disease in nonpregnant adults, 1990–2007.. Clin Infect Dis.

[42] Harrison LH, Dwyer DM, Johnson JA (1995). Emergence of serotype V group B streptococcal infection among infants and adults.. J Infect Dis.

[43] Blumberg HM, Stephens DS, Modansky M, Erwin M, Elliot J (1996). Invasive group B streptococcal disease: the emergence of serotype V.. J Infect Dis.

[44] van der Mee-Marquet N, Fourny L, Arnault L, Domelier AS, Salloum M (2008). Molecular characterization of human-colonizing *Streptococcus agalactiae* strains isolated from throat, skin, anal margin and genital body sites.. J Clin Microbiol.

[45] Suttle CA, Viruses in the sea (2005). Nature.

[46] Suttle CA (2007). Marine viruses-major players in the global ecosystem.. Nature Rev Microbiol.

[47] Lee CY, Iandolo JJ (1986). Integration of staphylococcal phage L54a occurs by site-specific recombination: Structural analysis of the attachment sites.. Proc Natl Acad Sci USA.

[48] Coleman D, Knights J, Russell R, Shanley D, Birkbeck TH (1991). Insertional inactivation of the *Staphylococcus aureus* beta-toxin by bacteriophage phi 13 occurs by site- and orientation-specific integration of the phi 13 genome.. Mol Microbiol.

[49] Chen Y, Golding I, Sawai S, Guo L, Cox EC (2005). Population fitness and the regulation of *Escherichia coli* genes by bacterial viruses.. PLoS Biol.

[50] Al Safadi R, Amor S, Hery-Arnaud G, Spellerberg B, Lanotte P (2010). Enhanced expression of lmb gene encoding laminin-binding protein in *Streptococcus agalactiae* strains harboring IS1548 in scpB-lmb intergenic region.. PLoS ONE.

